# Endogenous *n*-3 PUFAs Improve Non-Alcoholic Fatty Liver Disease through FFAR4-Mediated Gut–Liver Crosstalk

**DOI:** 10.3390/nu15030586

**Published:** 2023-01-22

**Authors:** Xuan Jiang, Qin Yang, Hongyan Qu, Yongquan Chen, Shenglong Zhu

**Affiliations:** 1 Wuxi School of Medicine, Jiangnan University, Wuxi 214122, China; 2 School of Food Science and Technology, Jiangnan University, Wuxi 214122, China; 3 Wuxi Translational Medicine Research Center and School of Translational Medicine, Jiangnan University, Wuxi 214122, China

**Keywords:** free fatty acid receptor 4, non-alcoholic fatty liver disease, gut–liver axis, *n*-3 polyunsaturated fatty acids

## Abstract

The gut–liver axis plays a key role in the development and progression of non-alcoholic fatty liver disease (NAFLD). Due to the complexity and incomplete understanding of the cross-talk between the gut and liver, effective therapeutic targets are largely unknown. Free fatty acid receptors (FFARs) may bridge the cross-talk between the gut and liver. FFAR4 has received considerable attention due to its important role in lipid metabolism. However, the role of FFAR4 in this cross talk in NAFLD remains unclear. In this study, mice with high endogenous *n*-3 PUFAs but FFAR4 deficiency were generated by crossbreeding Fat-1 and FFAR4 knockout mice. FFAR4 deficiency blocked the protective effects of high endogenous *n*-3 PUFAs on intestinal barrier dysfunction and hepatic steatosis. In addition, FFAR4 deficiency decreased gut microbiota diversity and enriched *Rikenella*, *Anaerotruncus*, and *Enterococcus*, and reduced *Dubosiella*, *Ruminococcaceae UCG-010*, *Ruminococcaceae UCG-014*, *Coriobacteriaceae UCG-002*, *Faecalibaculum*, *Ruminococcaceae UCG-009*, and *Akkermansia*. Notably, FFAR4 deficiency co-regulated pantothenic acid and CoA biosynthesis, β-alanine metabolism, and sphingolipid metabolism pathways in the gut and liver, potentially associated with the aggravation of NAFLD. Together, the beneficial effects of *n*-3 PUFAs on the gut and liver were mediated by FFAR4, providing insights on the role of FFAR4 in the treatment of NAFLD through the gut–liver axis.

## 1. Introduction

Non-alcoholic fatty liver disease (NAFLD) is characterized by metabolic dysfunction, ranging from hepatic steatosis and steatohepatitis to fibrosis [1]. Currently, no approved treatments are available despite the fact that NAFLD has become a worldwide epidemic [2,3]. The pathological mechanisms associated with the occurrence and progression of NAFLD are complex and remain largely unknown. A better understanding of the pathogenesis and identification of potential therapeutic targets of NAFLD can aid in the selection of optimal treatment options.

The gut–liver axis mediates the communication and feedback loop between the digestive system and the liver [4]. Research on the gut–liver axis provides new insights into the molecular mechanisms underlying the pathophysiology of NAFLD [5]. The gut microbiota serves as the connection point of the gut–liver axis, thus representing a potential target in NAFLD intervention [6,7]. Indeed, gut microbiota dysbiosis promotes the development of NAFLD by modulating the gut–liver axis, leading to increased gut permeability and consequent unrestricted transfer of microbial metabolites to the liver [8]. However, the effective targets are largely unknown due to the complexity of the crosstalk between the gut and liver.

G protein-coupled receptors are the most pharmacologically active and extensively studied targets [9,10]. The activation of free fatty acid receptors (FFARs) plays an important role in diverse biological processes [11], and they may bridge the cross-talk between the gut and liver. Among them, free fatty acid receptor 4 (FFAR4) has received much attention thanks to its role in the regulation of the beneficial effects of many long-chain fatty acids (LCFAs) on human health [12,13]. A study reported that *n*-3 polyunsaturated fatty acids (*n*-3 PUFAs) are involved in NAFLD by targeting the gut–liver axis [14]. They directly affect the composition and diversity of gut microbiota, contributing to the integrity of the intestinal mucosa and the improvement of intestinal permeability [15,16]. Interestingly, a study demonstrated that *n*-3 PUFAs may exert their functions independently of FFAR4 [17]. However, it is still unclear whether the beneficial effects of *n*-3 PUFAs on the gut–liver axis are mediated by FFAR4.

Therefore, this study evaluated the effects in vivo of endogenous fatty acids on the improvement of NAFLD through the gut–liver axis, and the results were compared between WT mice and FFAR4-deficient mice. Changes in intestinal barrier function, hepatic lipid, and functional enzyme markers, as well as glucose and insulin resistance, and hepatic steatosis in each group were assessed. In addition, an integrative analysis was performed on the gut microbiome and liver untargeted metabolome to explore the underlying mechanism used by FFAR4 to mediate gut–liver crosstalk.

## 2. Materials and Methods

### 2.1. Animal Experiments

Male wild-type mice (6 weeks) were obtained from Jicui Yaokang Biotechnology Co., Ltd. (Jiangsu, Nanjing, China). C57BL/6J FFAR4^−/−^ knockout mice were originally generated by Shanghai Bioraylab, as previously described [18]. The genotype analysis of the C57BL/6J Fat-1 mice carrying the C. elegans FAT-1 enzyme, which converts *n*-6 to *n*-3 polyunsaturated fatty acids, was performed as instructed by Jackson Laboratory [19]. Genotype identification was performed with PCR primers specific to each genotype (Figure S1A,B). FFAR4^−/−^/Fat-1 mice and their littermates were generated by crossbreeding FFAR4 knockout and Fat-1 mice. All animal procedures were conducted in accordance with the appropriate institutions and regulations and were approved by the Jiangnan University Animal Care and Use Committee (JN.No20210530c0601230[153]). The mice were randomly divided into several groups (*n* = 7–8, per group) according to the experimental needs: wild-type (WT)-HFD, Fat-1-HFD, FFAR4^−/−^-HFD, and FFAR4^−/−^/Fat-1-HFD groups were fed a high-fat diet (HFD, 60 kcal% fat, TP23300, TROPHIC, Nantong, China), and the WT-ND and Fat-1-ND group were fed a normal diet (ND, 10 kcal% fat, TP23302, TROPHIC, Nantong, China). After 16 weeks of high-fat or normal diet feeding, mice were sacrificed under anesthesia, and liver and adipose tissue were weighed. Mouse serum, feces, and tissue samples were collected and stored at −80 °C until further analysis. The area of epididymal white adipose tissue (eWAT), inguinal white adipose tissue (iWAT), and brown adipocytes (BATs) was measured using CT imaging.

### 2.2. Glucose and Insulin Tolerance Tests

The tolerance test for glucose (GTT) and insulin (ITT) was applied to mice fed a HFD at week 16. The mice were fasted for 6 h before the GTT or 4 h before the ITT and treated with an intraperitoneal (ip) injection of glucose (1.5 g/kg) or insulin (0.75 U/kg). The glucose concentration in the blood was measured using a glucometer (Accu-check, Roche Diagnostics) at 0 min (before injection) and at 15, 30, 90, and 120 min post-injection. The area under the curve was calculated using the trapezoidal method.

### 2.3. Biochemical Analysis

Hepatic total cholesterol (TC), triglycerides (TG), alanine aminotransferase (ALT), and aspartate aminotransferase (AST) were determined using the corresponding kits from Nanjing Jiancheng Bioengineering Institute (Nanjing, China).

### 2.4. Hematoxylin and Eosin Staining (H&E)

Fresh liver and colon tissue samples were collected, fixed in 4% paraformaldehyde in phosphate-buffered saline for 24 h, and embedded in paraffin. Tissue sections were stained using a standard H&E protocol. Stained sections were scanned using a digital slide scanner (3D HISTECH Ltd., Budapest, Hungary) and analyzed using ImageJ software. 

### 2.5. Fatty Acid Analysis

Fatty acid composition was determined by gas liquid chromatography as previously described [20]. In brief, total fatty acids in tissues and serum were extracted with chloroform. The fatty acids were then saponified and methyl esterified to produce fatty acid methyl esters, which were detected using the Q Exactive GC Orbitrap GC-MS/MS high resolution mass spectrometer (Trace1300, Thermo Fisher Scientific, Waltham, MA, USA). The capillary column of Rtx Wax column was used to separate fatty acids (30.0 m × 0.25 mm, Restek, Bellefonte, PA, USA). The entire detection time for all fatty acid peaks was approximately 35 min. Peaks were identified by the retention times of fatty acid methyl ester standards (Sigma-Aldrich, Saint Louis, MO, USA) using the Xcalibur acquisition software (version 4.0, Thermo Fisher Scientific, Waltham, MA, USA). Pentadecanoic acid (C15:0) was used as an internal standard and the percent composition of each fatty acid was calculated. 

### 2.6. Untargeted Metabolomics Analysis

Liver metabolites were analyzed as previously described [21]. In brief, compound extraction from liver samples (50 mg) was performed using a mixture of methanol/acetonitrile/deionized water (2:1:1, *v/v/v*), and then centrifuged at 12,000× *g*, 4 °C for 20 min. The supernatant (500 μL) was concentrated using a vacuum centrifugal concentration dryer for 4 h and re-suspended in 150 μL methanol/deionized water (4:1, *v/v*) before the analysis. Instrumental analysis was performed using an ultra-performance liquid chromatography and Q-Exactive high-resolution mass spectrometer system (Thermo Fisher Scientific, Waltham, MA, USA) with a C18 column (Waters Corporation, Milford, MA, USA). Compound Discover 3.2 software (Thermo Fisher Scientific, Waltham, MA, USA) was used for metabolite extraction, quantification, and relative abundance analysis of raw measurement data. The principal component analysis (PCA) and orthogonal partial least-squares discriminant analysis (OPLS-DA) models were conducted to identify the metabolic features indicating the difference between the Fat-1-HFD and FFAR4^−/−^/Fat-1-HFD groups. Metabolic features with variable importance in projection (VIP > 1 and *p* < 0.05) were considered potential differential biomarkers. Metabolic pathway analysis based on the identified metabolites was performed using MetaboAnalyst 5.0 (https://www.metaboanalyst.ca/; accessed on 26 September 2022). 

### 2.7. qRCR Analysis

For qPCR analysis, total RNA was extracted using the MolPure^®^ TRIeasy™ Plus Total RNA Kit (19211ES60, Yeasen, Shanghai, China), following the manufacturer’s instructions. RNA samples (1 µg) were reverse transcribed using HiScript III-RT SuperMix (R323-01, Vazyme, Nanjing, China) to generate cDNA. qPCR was performed using 2 × RealStar Power SYBR qPCR Mix (A313-01, GenStar, Beijing, China) on a RT-PCR system (CFX96 Real-Time PCR Detection System, Bio-Rad, Harales, CA, USA) using β-actin as a housekeeping gene. Relative changes in gene expression were calculated by the 2^−∆∆Ct^ method. All primer sequences are presented in Table S1.

### 2.8. Gut Microbiota Analysis 

DNA was extracted from the fecal samples using the FastDNA^®^Spin Kit for Feces (116570200, MP Biomedicals, Santa Ana, CA, USA). Samples were sequenced using previously described methods [22]. In brief, gut microbiota was analyzed by sequencing the V3–V4 region of 16S rRNA genes using the Illumina Miseq platform, according to the manufacturer’s instructions. The quantitative Insights into Microbial Ecology (QIIME2) platform was performed for 16S rRNA gene sequencing analysis. The α-diversity, β-diversity, and linear discriminant analysis (LDA) effect size (LEfSe) analyses were performed using Microbiomeanalyst (https://www.microbiomeanalyst.ca/; accessed on 28 September 2022). The metabolic functional characteristics of gut microbial communities were predicted using Phylogenetic Investigation of Communities by Reconstruction of Unobserved States (PICRUSt).

### 2.9. Statistical Analysis

Statistical analysis was performed using GraphPad Prism 8.0 software (GraphPad, San Diego, CA, USA) and SPSS 21.0 software (IBM Corporation, Armonk, NY, USA). All results are presented as mean ± SD. Student’s *t*-test and one-way ANOVA were used to compare two or multiple groups. A value of *p* < 0.05 was considered statistically significant.

## 3. Results

### 3.1. Endogenous n-3 PUFA Enrichment Improves HFD-Induced Intestinal Barrier Dysfunction and Hepatic Steatosis

**Figure 1:**
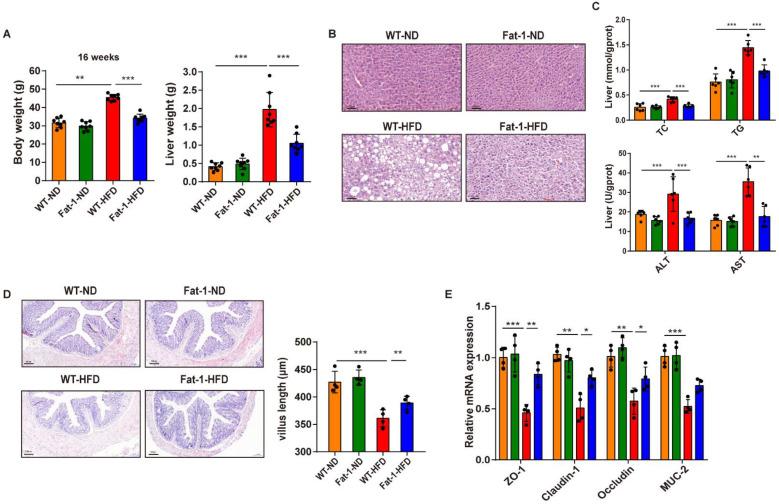
Endogenous *n*-3 PUFAs improve HFD-induced intestinal barrier dysfunction and hepatic steatosis. (**A**) Final body weight and liver weight at 16 weeks. (**B**) Liver pathology of different groups (200× magnification; scale bar = 50 μm). (**C**) Hepatic TC, TG, ALT, and AST content. (**D**) Representative images of H&E-stained colon sections (100× magnification; scale bar = 100 μm). Villus length quantified using ImageJ software (right). (**E**) qPCR analysis of genes involved in the intestinal barrier function. Results are presented as mean ± SD (*n* = 8 for each group). * *p* < 0.05, ** *p* < 0.01, and *** *p* < 0.001 by one-way ANOVA and Student’s *t*-test.

Fat-1 mice remarkably resisted the HFD-induced obesity compared with the WT mice. This may be due to the decrease in the *n*-6/*n*-3 PUFA ratio in the serum and the colon (Figure S1C). Furthermore, qPCR analysis was performed to identify the mRNA expression of FFAR4 in the colon of each group. The mRNA expression of FFAR4 in the colon was significantly downregulated in the WT-HFD group and was significantly increased by endogenous *n*-3 PUFAs (Fat-1-HFD group) (Figure S1D). This result indicated that high levels of endogenous *n*-3 PUFAs in the colon could activate the expression of FFAR4. Moreover, endogenous *n*-3 PUFAs attenuated HFD-induced glucose disorders and weight gain (Figure 1A, Figure S1E, and Figure S2A). They also significantly improved liver lesions and remarkably reduced the content of TC, TG, ALT, and AST (Figure 1B,C). Intestinal barrier function is critical for maintaining intestinal homeostasis, and high fat intake impairs the function of the intestinal barrier [23]. The length of the colon villus length and several intestinal barrier-related genes (*ZO-1*, *Claudin-1*, *Occludin*, and *MUC-2*) were significantly decreased due to the HFD, while the increase in genetic-induced *n*-3 fatty acids (in the Fat-1 transgenic mice) enhanced the expression of the above genes (Figure 1D,E). Collectively, these results showed that endogenous *n*-3 PUFAs contributed to the restoration of HFD-induced intestinal barrier dysfunction and hepatic steatosis. 

### 3.2. FFAR4 Deficiency Blocks Endogenous n-3 PUFAs Mediating the Improvement of Gut and Liver Functions

**Figure 2:**
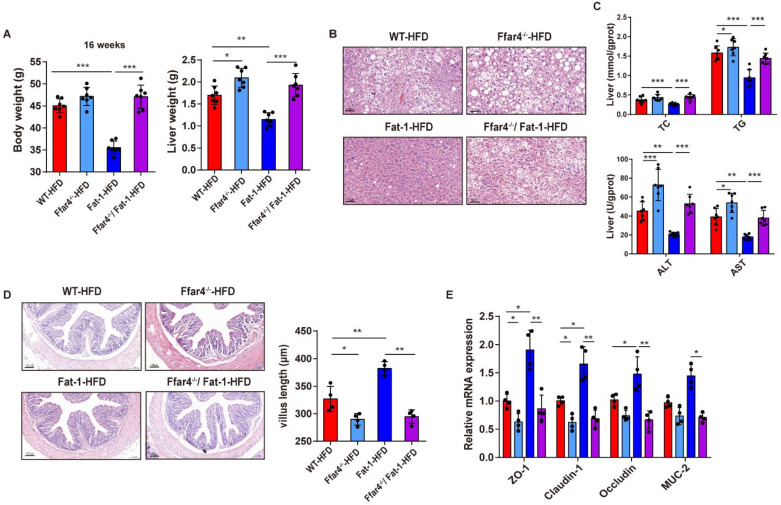
FFAR4 deficiency blocks the beneficial effects of endogenous *n*-3 PUFA. (**A**) Final body weight and liver weight at 16 weeks. (**B**) Liver pathology of different groups (200× magnification; scale bar = 50 μm). (**C**) Hepatic TC, TG, ALT, and AST content. (**D**) Representative images of H&E-stained colon sections (100× magnification; scale bar = 100 μm). Villus length quantified using ImageJ software (right). (**E**) Intestinal barrier function genes analyzed with qPCR. Results are presented as mean ± SD (*n* = 7, per group). * *p* < 0.05, ** *p* < 0.01, and *** *p* < 0.001.

FFAR4^−/−^/Fat-1 mice fed with a high-fat diet were used to further explore whether FFAR4 was involved in the improvement of gut and liver functions under NAFLD by endogenous *n*-3 PUFAs. FFAR4 deficiency blocked the beneficial effect of endogenous *n*-3 PUFAs on body weight, organ weight (liver and adipose tissue), and glucose metabolism (Figure 2A, Figure S1F, and Figure S2B). In addition, H&E section staining and determination of liver biochemical indicators were used to evaluate the effect of FFAR4 deficiency on liver pathology. FFAR4 deficiency suppressed the beneficial effects of endogenous fatty acids on liver pathology, as demonstrated by the aggravated hepatic fat vacuoles and the increased liver lipids (TC and TG) and injury markers (ALT and AST) as shown in Figure 2B,C. Furthermore, FFAR4 deficiency attenuated the improvement of endogenous *n*-3 PUFAs on the function of the intestinal barrier as determined by measuring the length of colonic villi and detecting the expression levels of intestinal barrier function genes by qPCR (Figure 2D,E). In summary, these results suggested that the improvement of gut and liver functions mediated by endogenous *n*-3 PUFAs required the participation of FFAR4.

### 3.3. FFAR4 Deficiency Alters the Diversity of Gut Microbiota in Fat-1 Transgenic Mice 

**Figure 3:**
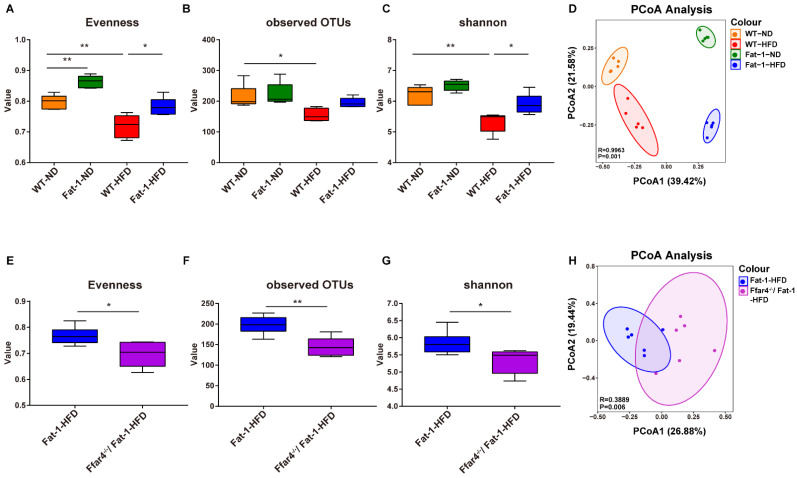
Diversity analysis of gut microbiota among different groups. Alpha diversity of the gut microbiome shown as evenness (**A**), observed OTUs (**B**), and Shannon index (**C**) among the WT-ND, Fat-1-ND, WT-HFD, and Fat-1-HFD groups. (**D**) PCoa plot of beta diversity in four groups (WT-ND, Fat-1-ND, WT-HFD, and Fat-1-HFD group). Alpha diversity of gut microbiome shown as evenness (**E**), observed OTUs (**F**), and Shannon index (**G**) between the Fat-1-HFD and FFAR4^−/−/^Fat-1-HFD groups. (**H**) PCoa of beta diversity in two groups (Fat-1-HFD and FFAR4^−/−^/Fat-1-HFD). Results are shown as mean ± SD (*n* = 5–6 for each group). * *p* < 0.05, ** *p* < 0.01.

The integrity of the intestinal barrier is closely related to the composition of gut microbiota [24]. Next, we assessed the effect of FFAR4 deficiency on the diversity of gut microbiota based on the 16S rRNA gene sequencing analysis. Endogenous *n*-3 PUFAs notably reversed the HFD-induced decrease in α-diversity, characterized by the increase in evenness, the number of observed operational taxonomic units (OTUs), and the Shannon index (Figure 3A–C). Furthermore, the difference in β-diversity among the four groups (WT-ND, Fat-1-ND, WT-HFD, and Fat-1-HFD) was clearly highlighted by the principal coordinates analysis (PCoA) (Figure 3D). However, FFAR4 deficiency abolished the effect of high endogenous *n*-3 PUFAs in restoring gut microbiota diversity (Figure 3E–H). 

### 3.4. FFAR4 Deficiency Alters Gut Microbiota Composition and Microbial Function Pathway in Fat-1 Transgenic Mice 

**Figure 4:**
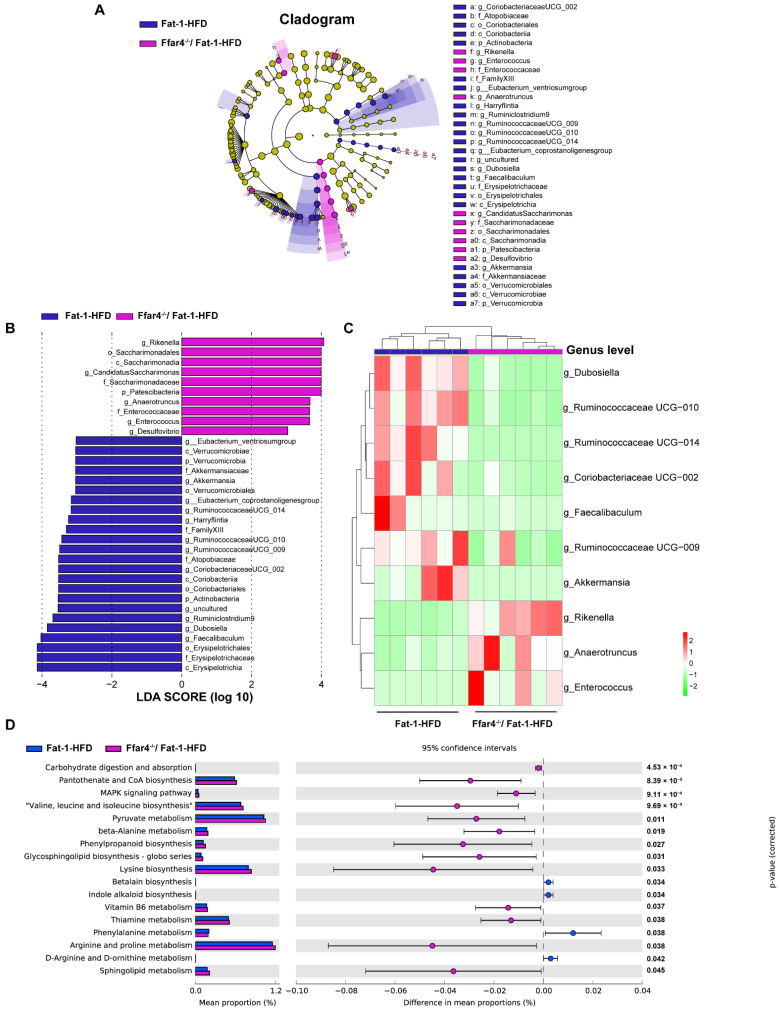
Gut microbial composition and microbial functional pathways. (**A**) Linear Discriminant Analysis (LDA) effect size (LEfSe) analysis between the Fat-1-HFD and FFAR4^−/−^/Fat-1-HFD groups (LDA score > 2 and *p* < 0.05). (**B**) Taxonomic cladogram was performed to identify the differentially abundant taxa. (**C**) Heat map of the differentially genus level (Top 10) between two groups. (**D**) Differential pathways between the Fat-1-HFD and FFAR4^−/−^/Fat-1-HFD groups (*p* < 0.05). Results are presented as mean ± SD (*n* = 6 for each group).

LEfSe analysis was conducted to identify the intestinal microbial biomarkers affected by FFAR4 deficiency (Figure 4A). Eighteen genera were identified as microbial biomarkers between the Fat-1-HFD and FFAR4^−/−^/Fat-1-HFD mice, as shown in Figure 4B. Moreover, the heatmap analysis revealed the top 10 significantly changed genera between the two groups (*p* < 0.05, Figure 4C). The FFAR4^−/−^/Fat-1-HFD group had an evident lower abundance of *Dubosiella*, *Ruminococcaceae UCG-010*, *Ruminococcaceae UCG-014*, *Coriobacteriaceae UCG-002*, *Faecalibaculum*, *Ruminococcaceae UCG-009*, and *Akkermansia* and a higher abundance of *Rikenella*, *Anaerotruncus*, and *Enterococcus* than that of the Fat-1-HFD group. Furthermore, a PICRUSt prediction analysis was performed to investigate the effect of FFAR4 deficiency on the metagenomic function of the gut microbiota following endogenous *n*-3 PUFAs enrichment. The 17 remarkably different pathways were identified between the two groups (*p* < 0.001–0.05, Figure 4D). Carbohydrate digestion and absorption, pantothenate and CoA biosynthesis, and the MAPK signaling pathway as well as valine, leucine, and leucine biosynthesis were significantly upregulated due to FFAR4 deficiency (*p* < 0.001). Therefore, these results indicated that FFAR4 deficiency altered the gut microbiota composition and microbial function pathway after endogenous *n*-3 PUFAs increase.

### 3.5. FFAR4 Deficiency Alters Hepatic Metabolites and Metabolic Pathways in Fat-1 Transgenic Mice 

**Figure 5:**
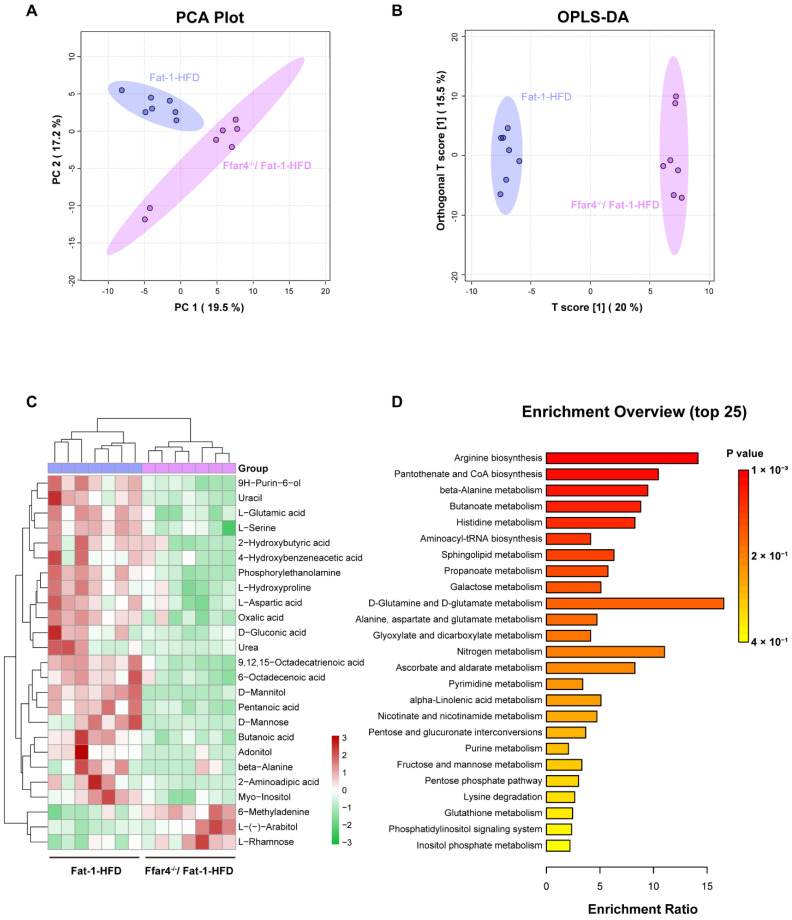
**Liver metabolomic analysis.** (**A**) PCA score plot. (**B**) OPLS-DA score plot. (**C**) Heat maps of differential metabolites between the Fat-1-HFD and FFAR4^−/−^/Fat-1-HFD groups, as determined by VIP > 1 and *p* < 0.05. (**D**) Overview of the metabolite set enrichment analysis. Results are shown as mean ± SD (*n* = 7, per group).

Am untargeted metabolomic analysis was conducted to further analyze the role of FFAR4 deficiency on liver metabolites in Fat-1 transgenic mice. The overall metabolite differences between the two groups were discriminated using PCA and OPLS-DA model analyses. The results demonstrated a significant difference in the distribution of metabolites between the Fat-1-HFD and FFAR4^−/−^/Fat-1 groups (Figure 5A,B). Moreover, 25 significantly different metabolites were found (VIP > 1, *p* < 0.05, Figure 5C). Next, metabolic pathway enrichment analysis was performed on the differential metabolites between the two groups to identify significantly altered metabolic pathways affected by FFAR4 deficiency. The overview of enrichment metabolic pathway analysis indicated that the metabolites significantly changed due to FFAR4 deficiency were involved in the 25 metabolic pathways (*p* < 0.05), with arginine biosynthesis being the most significantly changed pathway (Figure 5D). In all, these results suggest that FFAR4 deficiency altered liver metabolites and metabolic pathways in Fat-1 mice.

### 3.6. Cross Talk between Differential Liver Metabolites and Gut Microbiota

**Figure 6:**
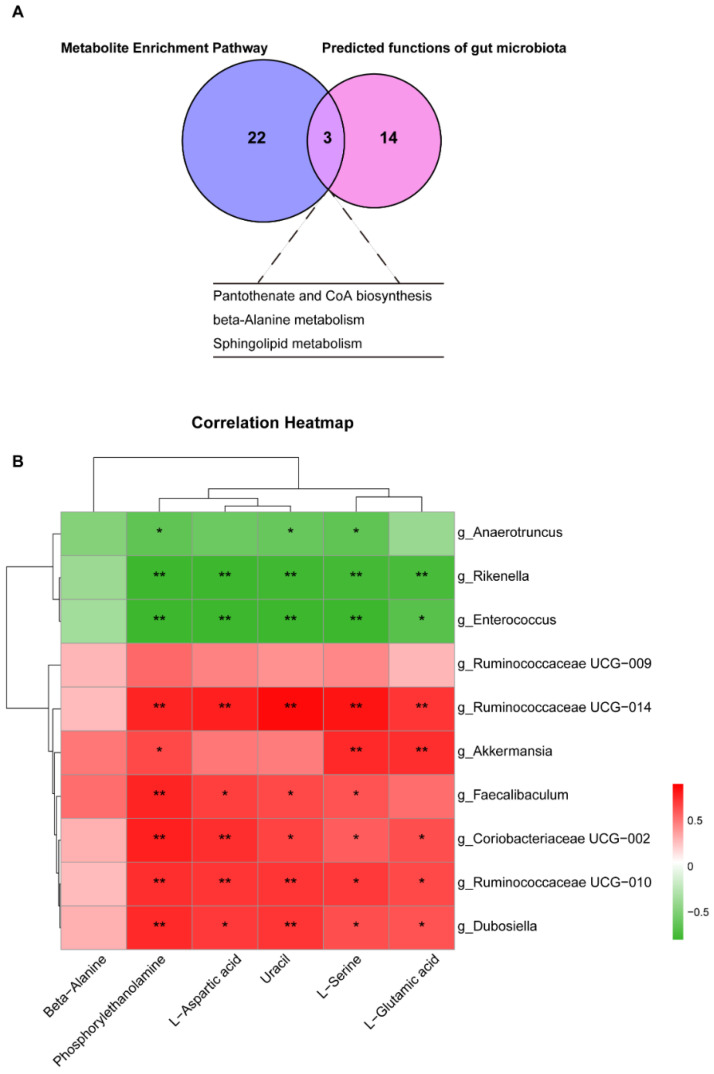
Key liver metabolites and gut microbiota affected by FFAR4. (**A**) Venn diagram analysis. (**B**) Correlation analysis of differential bacterial genera and metabolites. * *p* < 0.05 and ** *p* < 0.01.

A Venn plot intersection analysis was performed to identify common pathways between liver metabolite-increased pathways and predicted gut microbial functional pathways (Figure 6A). Pantothenate and CoA biosynthesis, beta-alanine metabolism, and sphingolipid metabolism were the most common differential metabolic pathways in the liver and gut. Additionally, we performed a correlation analysis to investigate the relationship between gut microbial biomarkers and liver differential metabolites belonging to three metabolic pathways. As shown in Figure 6B, the relative abundance of *Dubosiella*, *Ruminococcaceae UCG-010*, *Ruminococcaceae UCG-014*, *Coriobacteriaceae UCG-002*, *Faecalibaculum*, *Ruminococcaceae UCG-009*, and *Akkermansia* was positively associated with beta-alanine, phosohorylethanolamine, L-aspartic acid, uracil, L-serine, and L-glutamic acid content in the liver. However, the relative abundance of *Rikenella*, *Anaerotruncus*, and *Enterococcus* was negatively associated with the content of the above hepatic metabolites.

## 4. Discussion

**Figure 7:**
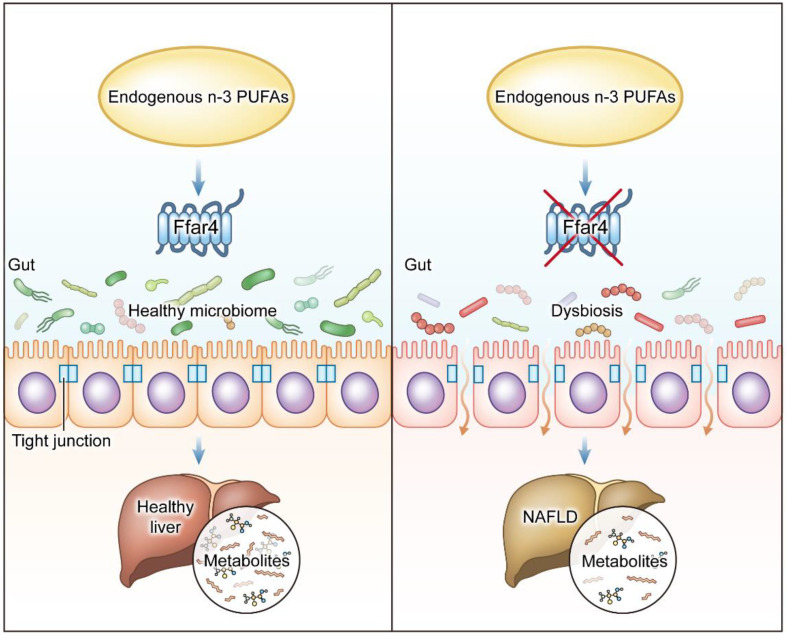
Endogenous *n*-3 PUFAs improved NAFLD through FFAR4-mediated gut–liver axis.

The increasing prevalence and global health burden of NAFLD have led to focused research and increased interest in the treatment of patients with NAFLD. Although many pathophysiological mechanisms and genetic variants of NAFLD have been identified, there are still no very effective drugs approved for the treatment of NAFLD. New drugs targeting the gut–liver axis have great potential in the treatment of NAFLD. Our results demonstrate that FFAR4 played an indispensable role in regulating the endogenous *n*-3 PUFA-mediated gut–liver axis to improve NAFLD (Figure 7).

Previous studies reported several conflicting results on the effect of *n*-3 PUFAs on NAFLD [25,26,27,28], without any clear explanation. The conflicting conclusions may be attributed to the genetic polymorphism of FFAR4 among populations, since several FFAR4 mutations exist among populations, as previously reported [29]. Therefore, LCFAs may influence NAFLD in a different manner in these populations. Furthermore, another possibility is agonist selectivity. The majority of FFAR4 agonists can also activate FFAR1, which is engaged in metabolic regulation [30]. However, FFAR1 is mainly located in the pancreatic β-cells and contributes to insulin secretion [31], and FFAR4 is expressed in metabolic organs such as the gut, adipose tissue, and liver [32]. Therefore, FFAR4 is probably working as a receptor involved in *n*-3 PUFAs regulating the gut–liver axis. In addition, numerous LCFAs bind to FFAR4, producing different results since fatty acids have different functions [33]. A clinical study shows that EPA and DHA can be used to improve blood lipid and inflammatory status in patients with hypercholesterolemia, while α-linolenic acid does not show such effect [34]. Our suggestion according to our results is that the protection of the gut and liver in NAFLD mediated by the increase in endogenous *n*-3 PUFAs was dependent on the FFAR4 signaling pathway. However, this study does not clarify whether the FFAR4 signal is more important than the *n*-6/*n*-3 PUFA ratio.

Clinical experimental studies on *n*-3 PUFA dietary supplements (fish oil) showed that *n*-3 PUFAs have beneficial effects on the liver in NAFLD patients by preventing lipid accumulation, reducing plasma triglyceride levels, and increasing insulin sensitivity [35,36]. Additionally, dietary *n*-3 PUFAs (EPA and DHA) inhibit intestinal barrier dysfunction by upregulating tight junction gene expression and reducing the secretion of inflammatory cytokines [37,38]. A study reported that NAFLD patients generally had a high dietary intake of *n*-6 PUFAs with an unbalanced *n*-6/*n*-3 PUFA ratio. Reducing the *n*-6/*n*-3 PUFA ratio and increasing *n*-3 PUFA intake can significantly improve NAFLD [39]. Supplementation of *n*-3 PUFAs exerts a variety of health benefits on the human body by activating the FFAR4 signaling pathway [40]. In this study, our results showed that FFAR4 knockout blocked the protective effect of *n*-3 PUFAs on intestinal barrier function. Maintenance of intestinal barrier function is essential for intestinal health and homeostasis. The dysfunction of the intestinal barrier is tightly associated with the dysbiosis of gut microbiota, leading to increased intestinal permeability and unrestricted transport of microbial metabolites to the liver, thereby exacerbating the development of NAFLD [41,42]. The reduction in gut microbiota diversity was directly proportional to the severity of NAFLD. *n*-3 PUFAs improve HFD-induced gut microbiota dysbiosis by increasing the abundance of beneficial bacteria such as *Bifidobacterium*, *Lactobacillus*, *Akkermansia*, and butyrate-producing bacteria [43,44,45]. Here, we performed a comprehensive analysis of the gut microbiota using 16S rRNA amplicon sequencing. This study revealed that FFAR4 deficiency inhibited the beneficial effects of *n*-3 PUFAs on the composition and diversity of gut microbiota, with *Dubosiella*, *Ruminococcaceae UCG-010*, *Ruminococcaceae UCG-014*, *Coriobacteriaceae UCG-002*, *Faecalibaculum*, *Ruminococcaceae UCG-009*, *Akkermansia*, *Rikenella*, *Anaerotruncus*, and *Enterococcus* as the core bacterial genera affected by FFAR4 deficiency. *Dubosiella* was a potentially beneficial bacterium, and its relative abundance changes might be involved in lipid metabolism [46]. *Ruminococcaceae* and *Faecacterium* were the major producers of short-chain fatty acids (SCFA) and participated in the prevention of metabolic syndrome and intestinal diseases [47,48]. *Akkermansia muciniphila* was thought to be critical for the maintenance of mucus layer integrity [49]. The high abundance of *Rikenella* was associated with the induction of infectious colitis and colorectal cancer [50]. The relative abundance of *Anaerotruncus* generally increased in obese mice and was positively correlated with glucose intolerance [51]. *Enterococcus* was identified as a major conditional pathogen responsible for liver inflammation and disease progression [52]. These studies indicated that FFAR4 deficiency caused a decrease in the abundance of beneficial bacteria with regulated lipid metabolism, producing SCFAs, reducing inflammation, and protecting intestinal barrier function, while upregulating the abundance of harmful bacteria that promote inflammation and glucose metabolism disorders. Therefore, we pointed out that FFAR4 deficiency has abolished the effect of *n*-3 PUFAs on improving the diversity and composition of the gut microbiota, characterized by a decrease in the abundance of genera that contribute to NAFLD prevention while increasing the abundance of genera that promote NAFLD. However, the specific mechanism by which FFAR4 affects the abundance of these bacteria requires further experimental exploration. 

Microbiota-derived metabolites contribute to the maintenance of liver energy homeostasis. Liver metabolites are altered with changes in gut microbiota, demonstrating the etiological mechanism of NAFLD [53]. Moreover, gut microbiota can directly participate in the absorption, bioavailability, and biotransformation of fatty acids [54]. PCA and OPLS-DA plots demonstrated a clear distinction in the spatial distribution of the overall composition of liver metabolites between the two groups. Pathway enrichment analysis of differential metabolites in the liver indicated that FFAR4 deficiency mainly affected amino acid and fatty acid metabolism pathways. Additionally, the joint analysis of multi-omics (microbiomics and metabolomics) can more accurately evaluate the mechanism regulating the crosstalk between the gut and liver. Our results suggested that metabolites and gut microbiota involved in pantothenate and CoA biosynthesis, beta-alanine metabolism, and sphingolipid metabolism might play a key role in the FFAR4-mediated communication of the gut–liver axis. The gut–liver axis is a bidirectional interaction between gut microbiota and liver metabolites. Metabolites secreted by the liver may affect the composition of gut microbiota as endocrine signaling molecules. Our research used systemic knockout mice, and it is uncertain whether the loss of FFAR4 in the liver can affect intestinal function. Generating tissue-specific conditional knockout mice may contribute to addressing this issue.

## 5. Conclusions

In conclusion, our results indicated that the beneficial effects of endogenous n-3 PUFAs on metabolic disorders, which were mediated by Ffar4. Gut microbiota and metabolites are involved in gut–liver axis communication. Furthermore, metabolites and bacteria involved in pantothenate and CoA biosynthesis, beta-alanine metabolism, and sphingolipid metabolism work as crucial mediators for the *n*-3 PUFAs-FFAR4 pathway. The findings of this study highlight the underlying mechanism of FFAR4-mediated gut–liver crosstalk involved in the occurrence of NALFD and provide new ideas for the study of FFAR4 as a therapeutic target for NAFLD.

## Data Availability

Data supporting this study are available from the corresponding author upon reasonable request.

## Supplementary Materials

The following supporting information can be downloaded at: https://www.mdpi.com/article/10.3390/nu15030586/s1, Figure S1: Mice genotype identification and results of glucose tolerance and insulin tolerance tests on different groups of mice. (A) and (B) Identification of mouse genotypes. (C) The ratio of *n*-6/*n*-3 fatty acids in serum or colon tissue. (D) The mRNA expression of FFAR4 in the colon tissue. (E) and (F) Area under the curve (AUC) for GTT or ITT; Figure S2: Comparison of adipose tissue weight and distribution in each group of mice. (A) eWAT, iWAT, and BAT weight among four groups. (B) CT measurements of eWAT and iWAT. Weight and area are shown on the right; Table S1: qPCR primer sequences.
