# Supplementary material for: Endogenous n-3 PUFAs Improve Non-Alcoholic Fatty Liver Disease through FFAR4-Mediated Gut–Liver Crosstalk
Source: Nutrients. 2023 Jan 22;15(3):586. doi: 10.3390/nu15030586 (PMC9919706; doi:10.3390/nu15030586)
Supplement: Supplementary file 1 [file nutrients-15-00586-s001.zip › Supplementary Table S1.pdf]

Supplementary materials

Table S1. qPCR primer sequences

Gene name	Forward sequnence (5’→ 3’)	Reverse sequence (5’→ 3’)
Ffar4	CAACAAGACTACCGACTC	GATGAGGAGGATGGTGAT
ZO-1	GCTTTAGCGAACAGAAGGAGC	TTCATTTTTCCGAGACTTCACCA
Claudin-1	GCCTTGATGGTAATTGGCATCC	GGCCACTAATGTCGCCAGAC
Occludin	TTGAAAGTCCACCTCCTTACAGA	CCGGATAAAAAGAGTACGCTGG
MUC-2	AGGGCTCGGAACTCCAGAAA	CCAGGGAATCGGTAGACATCG
β-actin	GTGACGTTGACATCCGTAAAGA	GCCGGACTCATCGTACTCC
