# Supplementary figures and images for: Endogenous n-3 PUFAs Improve Non-Alcoholic Fatty Liver Disease through FFAR4-Mediated Gut–Liver Crosstalk
Source: Nutrients. 2023 Jan 22;15(3):586. doi: 10.3390/nu15030586 (PMC9919706; doi:10.3390/nu15030586)

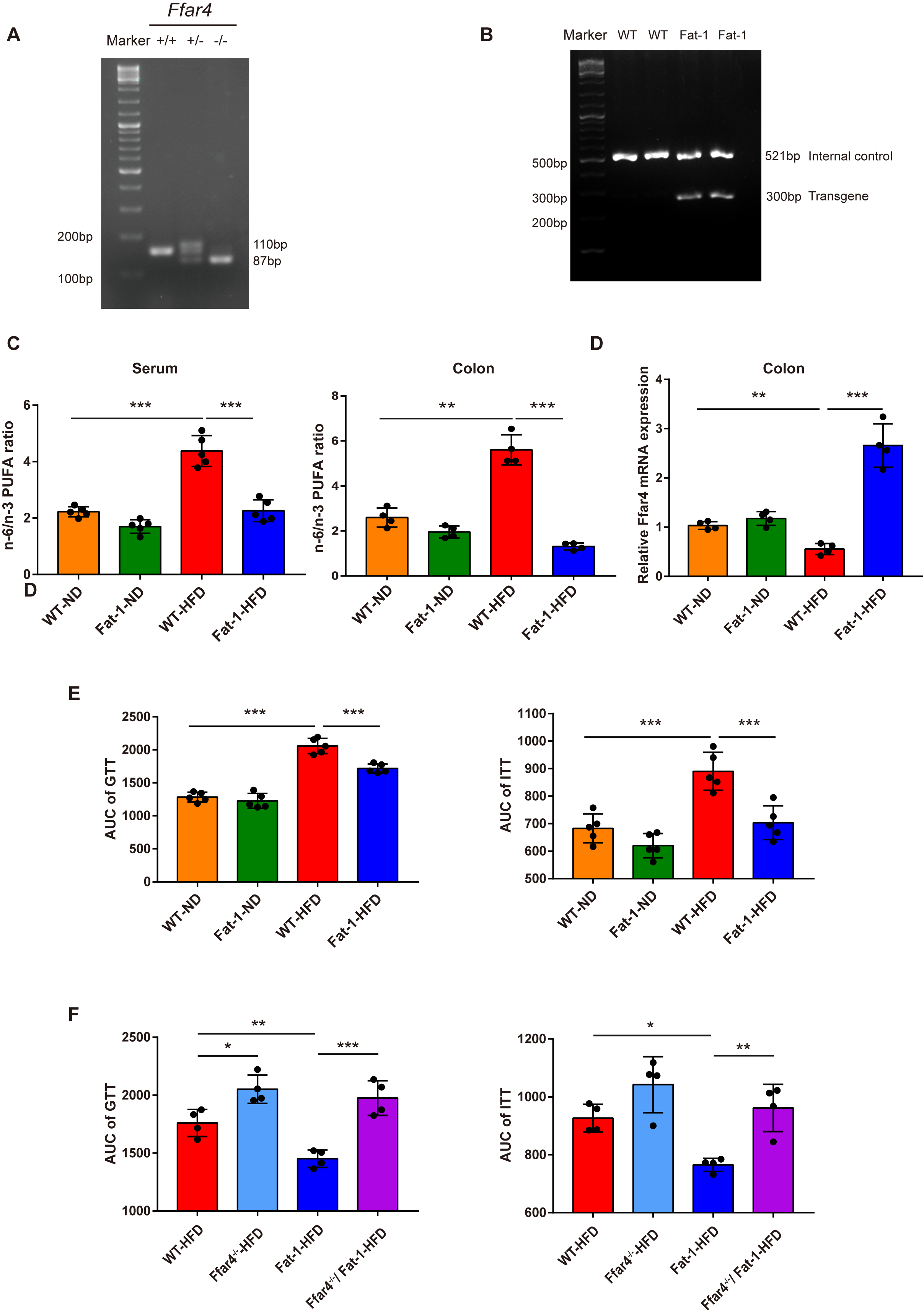

Supplement: Supplementary file 1 [file nutrients-15-00586-s001.zip › Figure S1.tif]

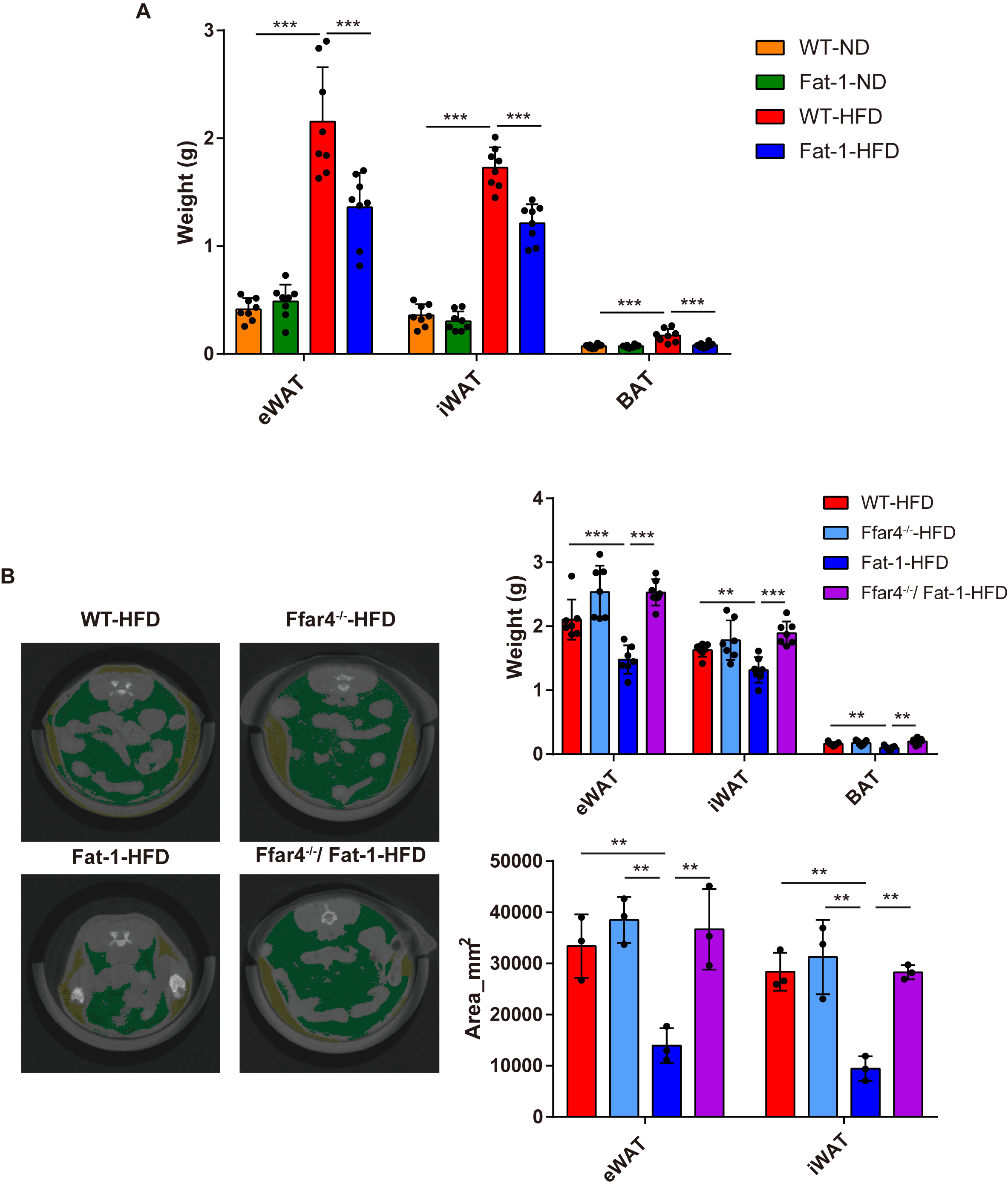

Supplement: Supplementary file 1 [file nutrients-15-00586-s001.zip › Figure S2..tif]
